# Caspase-8 contributes to an immuno-hot microenvironment by promoting phagocytosis via an ecto-calreticulin-dependent mechanism

**DOI:** 10.1186/s40164-022-00371-1

**Published:** 2023-01-12

**Authors:** Zhihua Gong, Qingzhu Jia, Jinming Guo, Chongyi Li, Shouxia Xu, Zheng Jin, Han Chu, Yisong Y. Wan, Bo Zhu, Yi Zhou

**Affiliations:** 1 Department of Oncology, Xinqiao Hospital, Army Medical University, Chongqing, 400037 China; 2 Chongqing Key Laboratory of Immunotherapy, Chongqing, 400037 China; 3 School of Pharmacy and Bioengineering, Chongqing University of Technology, Chongqing, 400054 China; 4 Department of Ophthalmology, Daping Hospital, Army Medical University, Chongqing, 400042 China; 5 GloriousMed Clinical Laboratory Co., Ltd, Shanghai, People’s Republic of China; 6 Center of Growth, Metabolism and Aging, Key Laboratory of Bio-Resources and Eco-Environment, College of Life Sciences, Sichuan University, Chengdu, 610064 China; 7 Department of Microbiology and Immunology, Lineberger Comprehensive Cancer Center, University of North Carolina at Chapel Hill, Chapel Hill, North Carolina 27599 USA

**Keywords:** Caspase-8, Calreticulin, Antigen presentation, Dendritic cells, Immunotherapy, Tumor microenvironment

## Abstract

**Background:**

Caspase-8 (Casp8) acts as an initiator in cell apoptosis signaling. However, the role of Casp8 in tuning the tumor immune microenvironment remains controversial due to the complicated crosstalk between immune-tolerogenic apoptotic cell death and immunogenic cell death cascades.

**Methods:**

The Cancer Genome Atlas (TCGA) and publicly accessible immune checkpoint blockade (ICB)-treated cohorts were used to investigate the clinical relevance of Casp8. A tumor-bearing mouse model was used to characterize changes in the tumor microenvironment and to explore the efficacy of ICB treatment under Casp8 knockout conditions.

**Results:**

By exploring TCGA datasets, we showed that the expression level of Casp8 was associated with an immuno-hot microenvironment across various solid tumor types. Casp8 deficiency leads to decreased CD8^+^ T cell infiltration and resistance to anti-PD-L1 therapy in a mouse model. Mechanistically, Casp8 deficiency or pharmacological disruption results in impaired ecto-calreticulin transition in tumor cells, which in turn hampers antigen presentation in draining lymph nodes. Furthermore, radiotherapy restored sensitivity to anti-PD-L1 treatment via elevated calreticulin surface expression.

**Conclusions:**

Our data revealed a causative role of Casp8 in modulating the immunogenicity of tumor cells and responsiveness to ICB immunotherapies and proposed radiotherapy as a salvage approach to overcome Casp8 deficiency-mediated ICB resistance.

**Supplementary Information:**

The online version contains supplementary material available at 10.1186/s40164-022-00371-1.

## Background

The application of immune checkpoint blockade (ICB), such as programmed death-1 receptor (PD-1) and programmed death ligand 1 (PD-L1) antibodies, was shown to reinvigorate T cell function and prolong survival in various cancer types [1–5]. However, due to intrinsic or acquired drug resistance, only a minority of patients experience long-term benefits from ICB. Pre-existing cytotoxic T cells in the tumor microenvironment (TME) are a prerequisite for the reinvigoration of T cells and the resulting inflammation, indicating that an immuno-hot TME induces a beneficial response to ICB administration [6]. However, the intrinsic tumor-driven force that leads to an immuno-hot TME remains unclear.

It is known that the form of tumor cell death instructs an immuno-hot or -cold TME. Immunogenic cell death (ICD) supplies an immuno-hot TME by promoting antigen release, antigen presentation, and cytotoxic T cell activation, inducing successful antitumor immunity [7]. Strategies aimed at eliciting ICD have been used to overcome resistance to ICB treatments [8]. Necroptosis, pyroptosis, and ferroptosis are the predominant immunogenic forms of cell death, and apoptosis is usually regarded as an immune-tolerogenic process [9–11]. Furthermore, cells that undergo necroptosis activate the immune system, particularly through antigen presentation and cross-priming of CD8^+^ T cells [12]. In pyroptosis, gasdermin proteins are cleaved by inflammatory caspases, leading to inflammatory cytokine release and cell death [13]. In cancer cells, gasdermin E cleaved by caspase-3 is an essential mediator of pyroptosis, which converts non-inflammatory apoptotic signals into pyroptotic cell death and suppresses tumor growth [14].

Caspase-8 (Casp8) is a switch for immune-tolerogenic apoptosis, immunogenic necroptosis, and pyroptosis [15]. It has been reported that in the presence of Casp8 malfunctions, the form of cell death could switch to necroptosis [16]. It is likely that Casp8 malfunction leads to ICD in cancer cells, which may provoke an adaptive immune response, facilitating CD8^+^ T cell infiltration and inducing an inflamed (hot) TME, which in turn improves the efficacy of ICB immunotherapies. Consistent with this theory, in a TRAF^−/−^ melanoma mouse model, tumor cells redirected the TNF signaling pathway to favor RIPK1-dependent necroptosis, enhance tumor eradication, and show a better response to anti-PD-1 therapy than the control group [17].

However, evidence also suggests that necrosis-induced inflammation only facilitates tissue repair responses and is not sufficiently effective to induce anticancer immunity [18, 19]. Moreover, the function of Casp8 may be catalytically activity-dependent or -independent. Apart from the aforementioned role of cleavage-dependent Casp8 function, the expression of catalytically inactive Casp8 is both necessary and sufficient to induce inflammasome formation [15]. This implies a complicated role of Casp8 in cell death and adaptive and innate immune responses.

In this study, we explored The Cancer Genome Atlas (TCGA) database and ICB-treated cohorts to determine the role of Casp8 in the TME and ICB responsiveness. We further established a Casp8 knockout cell line and animal models to understand the underlying mechanisms.

## Methods

### Cell lines

B16F10 cells were purchased from the American Type Culture Collection and cultured in Dulbecco’s modified Eagle’s medium supplemented with fetal bovine serum (10%), penicillin (100 U/mL), and streptomycin (100 mg/mL) at 37 °C in a humidified atmosphere containing 5% CO_2_. The caspase-8-knockout B16F10 cell line (B16-C8KO) was generated using CRISPR/Cas9 technology. The gRNAs encoding caspase-8 are shown in Additional file 1: Fig. S1.

### Animals and animal models

Female C57BL/6 mice aged 6–8 weeks were purchased from the Center of Experimental Animals of the Third Military Medical Univercity (TMMU). Nude mice were purchased from VitalStar Biotechnology Co. Ltd. (Beijing, China). The mouse handling protocols were approved by the Institutional Animal Care and Use Committee of TMMU. To establish tumor models, B16F10 cells (2 × 10^5^ in 100 µL of PBS) were subcutaneously inoculated into the right flank of 6–8-week-old female C57BL/6 mice or nude mice. When the tumors became palpable, tumor volume was monitored twice per week. In immunotherapeutic models, 2 × 10^5^ B16F10 cells were subcutaneously inoculated into the right flank of 6–8-week-old female C57BL/6 mice. Mice received 200 µg of intraperitoneal anti-PD-L1 monoclonal antibody (10F.9G2, Be0101, BioXcell) or the equivalent isotype control antibody (BioXcell, BE0090) on days 4, 7, and 10.

For radiation-combined immunotherapeutic models, 2 × 10^5^ B16F10 cells were subcutaneously inoculated into the right legs of C57BL/6 mice. When the tumors reached approximately 50 mm^3^, the mice were locally irradiated using the Varian Trilogy Stereotactic System at a single dose of 20 Gy. On the same day, 200 µg of anti-PD-1 monoclonal antibody (10F.9G2, Be0101, BioXcell) or equivalent isotype control antibody (BioXcell, BE0090) was injected intraperitoneally every three days three times.

### Detected cell surface ecto-calreticulin (ecto-CTR) expression and total calreticulin (total CRT) expression

The B16-C8KO cells or control cells (B16F10 cells treated with Z-IETD-FMK or dimethyl sulfoxide (DMSO) for 8 h) were collected and washed with PBS with 0.3% goat serum, fixed with 4% formaldehyde with 10% goat serum solution, and incubated with calreticulin antibody at 4 °C for 1 h. After washing three times with FACS buffer, ecto-CRT was detected using flow cytometry. To detect total CRT, the cells were fixed using a Cytofix/Cytoperm Kit (554714, BD). After washing twice with wash buffer, the samples were enclosed in 10% goat serum, incubated with the calreticulin antibody at 4 °C for 30 min, and detected by flow cytometry.

### In vivo phagocytosis assay

In vivo phagocytosis was performed in accordance with a previously established protocol [20]. Briefly, B16F10 cells were stained with 1 µM Cell Tracker Deep Red dye (Invitrogen), following the manufacturer’s protocol, and then treated with 50 µM Z-IETD-FMK or DMSO for 30 min, follow by 25 µM doxorubicin for 24 h. Cells were harvested and adjusted to 5 × 10^7^ cells/mL in PBS. Labeled tumor cells (5 × 10^6^ in 100 µL PBS) were injected into the spleen. After 2 h, the mice were sacrificed, the spleens were harvested and stained with anti-mouse CD11c antibody, and phagocytosis was assessed by flow cytometric analysis.

### Flow cytometry

In subcutaneous animal models, tumors were harvested on days 18–20. After euthanasia, the tumors were collected and filtered through a 70-μm cell strainer to obtain single-cell suspensions. For the analysis of tumor-infiltrating immune cells, the samples were stained with anti-CD45 (30-F11), anti-CD11b (M1/70), anti-CD3 (17A2), anti-CD8 (53-6.7), anti-CD4 (GK1.5), anti-F4/80 (BM8), anti-Gr-1 (RB6-8C5), and Fixable Viability Dye eFluor 780 (65-0865) (eBioscience). For T cell function analysis, samples were cultured with a cell stimulation cocktail (00-4975-03; eBioscience) for 6 h and subsequently stained with anti-CD45 (30-F11), anti-CD3 (17A2), anti-CD8 (53-6.7), anti-IFN-γ (XMG1.2), and anti-GZMB(GB11) using the Cytofix/Cytoperm™ Kit (554714, BD).

In the subcutaneous mouse model, draining lymph nodes were harvested for dendritic cell analysis. After filtering through a 70-μm cell strainer, the single-cell suspensions were stained with anti-CD11c (N418), anti-CD103 (2E7), anti-CD45 (30-F11), anti-MHC-II (M5/114.15.2), and Fixable Viability Dye eFluor 780 (65-0865) (eBioscience). Data were collected using a Gallios flow cytometer (Beckman Coulter) and analyzed using FlowJo software. To detect the cell surface and total calreticulin, cells were treated with the caspase-8 inhibitor Z-IETD-FMK or DMSO and then irradiated using the Varian Trilogy Stereotactic System with a single dose of 20 Gy. After 16 h, the cells were collected for analysis. Staining protocols were performed according to the manufacturer’s instructions, using anti-calreticulin (EPR3924, Abcam).

### Data collection

Bulk RNA sequencing data from 31 tumor types and four ICB-treated datasets with clinical information are available in the database of TCGA and under Accession codes: PRJEB23709 [21], GSE78220 [22], and GSE91061 [23]; see Table S8 of the original paper [24].

### RNA sequencing

Five pairs of control or B16-C8KO subcutaneous tumors from the right flank of 6–8-week-old female C57BL/6 mice were collected for RNA sequencing.

### Bioinformatics analysis

To investigate the contribution of caspase-8 to the inflamed TME, the cases were split into CASP8-high or CASP8-low groups based on the expression level of caspase-8 in each tumor type. The 1st and 4th quartiles were defined as CASP8-high and CASP8-low, respectively. Gene rank was generated based on the log2 fold change between the two groups, which was calculated using the R package *DESeq2* [25]. Then, the normalized enrichment scores and p-values of the inflamed TME gene set [26] were computed by *fgsea* for each tumor type. To confirm the significance, enrichment analysis was applied to the cases with group information using Gene Set Enrichment Analysis (GSEA). A heatmap was plotted based on the group information for each gene in the gene set.

To validate the benefit of caspase-8 for ICB treatment, cases in each ICB-treated cohort were grouped as CASP8-high and CASP8-low based on the median expression level of CASP8. The hazard ratio was calculated by *survival* and *survminer*, and forest plots were plotted using *forest plot*s. A heatmap was plotted for each gene of the inflamed TME gene set in ICB-treated datasets. Columns of the heatmap were arranged according to the expression level of CASP8.

To characterize the transcriptome profile of Casp8 knockout and control cells in B16-bearing mice principal component analysis was performed using *FactoMineR,* and the top two principal components were used for plotting. Enrichment signaling pathways were analyzed using Gene Ontology based on the differentially expressed genes between caspase-8-Casp8 knockout and control mice.

### Statistics

Comparisons between two groups of continuous variables were performed using an unpaired *t*-test or Mann–Whitney *U* test. Comparisons of continuous variables from three or more groups were performed using one-way analysis of variance (ANOVA). The association between responders and the categorical variables Casp8-high and Casp8-low was compared using the *χ*^2^ test or Fisher’s exact test. Tumor growth was compared using one-way ANOVA. Survival was estimated using Kaplan–Meier curves, and the p-value and hazard ratio were determined using a log-rank test. Statistical analyses were performed using Prism 6 software (GraphPad, Prism Software Inc., CA, USA) and R version 4.0.0. Statistical significance was set at p < 0.05.

## Results

### Caspase-8 indicates an immuno-hot TME in solid tumors

**Fig. 1:**
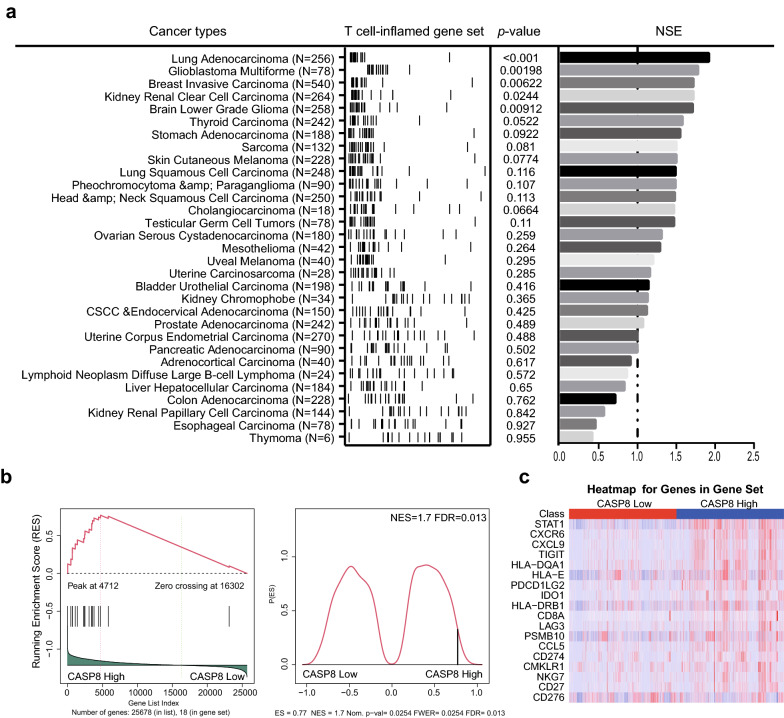
The correlation of CASP8 transcription and the 18 gene set ICB predictive biomarker. A total of 4874 patients across 31 cancer types from TCGA were enrolled for analysis. **a** GSEA comparing T cell-inflamed gene sets (measurement of ICB-responsiveness) between CASP8 -low and CASP8-high patients in TCGA. **b** Enrichment score of the T cell-inflamed gene set across 31 TCGA datasets. **c** Heatmap of the expression of 18 genes in the T cell-inflamed gene set in CASP8-low and CASP8-high patients. The 1st and 4th quartiles were defined as CASP8-low and CASP8-high, respectively

To investigate whether Casp-8 contributes to an inflamed TME favoring effective antitumor immunity, designated as immuno-hot TME in solid tumor types, expression profiling data from TCGA were introduced. A normalized GSEA score was used to compare the immuno-hot and -cold microenvironments between patients with high or low (1st and 4th quartiles as the cutoff for high vs. low groups) expression levels of CASP8 with a well-established T cell-inflamed gene set. For all 31 types of solid tumors (4874 cases) in TCGA, we observed consistently over-presented (normalized enrichment score > 0) T cell-inflamed gene sets in patients with high CASP8 expression levels, indicating an immuno-hot microenvironment in these patients (Fig. 1a). In addition, when applying this analysis to a merged cohort containing all the above tumor types, we confirmed the significant upregulation of the T cell-inflamed gene set in patients with high levels of CASP8 (Fig. 1b). Furthermore, the heatmap in Fig. 1c clearly illustrates a pattern of upregulated expression of 18 genes in the T cell-inflamed gene set, further supporting a strong association between CASP8 expression and the immuno-hot microenvironment.

### Knockout of Casp8 results in an immuno-cold microenvironment

To determine whether Casp8 expression is causative or merely a concomitant characteristic of an immuno-hot microenvironment, a B16-C8KO cell line was established using the CRISPR/Cas9 strategy. Western blotting and sequencing assays demonstrated the effectiveness of our depletion strategy at both the genomic and protein levels (Additional file 1: Fig. S1a and b). Consistently, a decrease in cell apoptosis was observed in vitro and in tumor-bearing immunocompetent mice (Additional file 1: Fig. S1c and d).

**Fig. 2:**
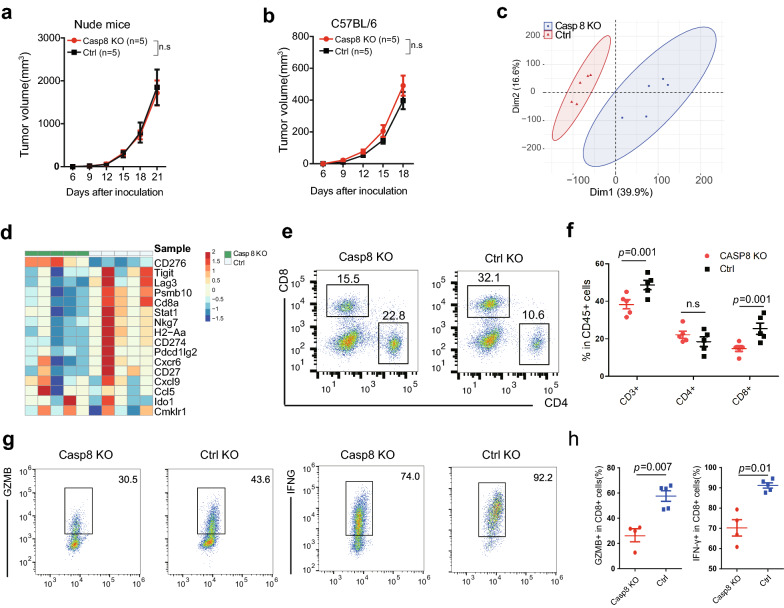
Casp8 knockout B16F10 tumors were associated with a cold TME. **a** B16-C8KO or control cells (2 × 10^5^ in 100 µL of PBS) were subcutaneously inoculated into the right flanks of 6–8-week-old nude mice. The tumor volume was monitored twice per week. **b** B16-C8KO or control cells (2 × 10^5^ in 100 µL of PBS) were subcutaneously inoculated into the right flanks of 6–8-week-old C57BL/6 mice. The tumor volume was monitored twice per week. **c** and **d** Analysis of the transcriptome of subcutaneous B16F10 tumors in C57BL/6 mice. **c** Unsupervised principal component analysis based on the 18 ICB responsiveness-related genes. **d** Heat map of the 18 genes. **e** and **f** B16-C8KO or control cells were subcutaneously inoculated into the right flanks of C57BL/6 mice, the TME of subcutaneous xenografts was analyzed. **e** Representative flow cytometry of CD4^+^ or CD8^+^ tumor-infiltrating cells. **f** Fractions of CD3^+^, CD4^+^, or CD8^+^ cells in CD45^+^ leukocytes in tumors. **g** and **h** Production of granzyme-B and INF-γ by CD8^+^ T cells was determined by flow cytometry. **g** Representative flow cytometry of tumor-infiltrating cytotoxic T cell function and **h** statistical analysis

To identify the immunological consequences of Casp8 knockout, B16-C8KO and control cells were subcutaneously inoculated into immunodeficient nude mice and immunocompetent mice. Identical tumor volumes were observed in immunodeficient nude mice, demonstrating no intrinsic difference in the cell growth rates resulting from Casp8 knockout (Fig. 2a). For the immunocompetent group, although there was no statistically significant difference in tumor size, we identified numerically increased tumor volumes in B16-C8KO-bearing mice, implicating an immuno-dependent mechanism of Casp8-mediated antitumor immunity (Fig. 2b). To comprehensively characterize the TME, homogenates of the tumor mass were subjected to RNA sequencing profiling. Principal component analysis revealed a pattern of fully distinct transcriptomes between B16-C8KO and control tumors, demonstrating a substantial reprogramming of the TME due to Casp8 knockout (Fig. 2c). Focusing on a list of prominent genes in the antitumor immune response, a trend of lower expression of these genes was found in the B16-C8KO group (Fig. 2d), which successfully recapitulated our observations in the TCGA cohort (Fig. 1c).

Furthermore, flow cytometry showed significantly impaired infiltration of T cells, especially CD8^+^ T cells, in the B16-C8KO microenvironment (Figs. 2e and f and Additional file 1: Fig. S2a). Profiling analysis revealed comparable infiltration of myeloid-derived suppressor cells and tumor-associated macrophages (Additional file 1: Fig. S3). Moreover, regarding the cytotoxic function of CD8^+^ T cells, granzyme-B and INF-γ production decreased in B16-C8KO tumor cells (Figs. 2g and h and Additional file 1: Fig. S2b).

### Poor responsiveness to anti-PD-L1 treatment in the B16-C8KO-bearing model

**Fig. 3:**
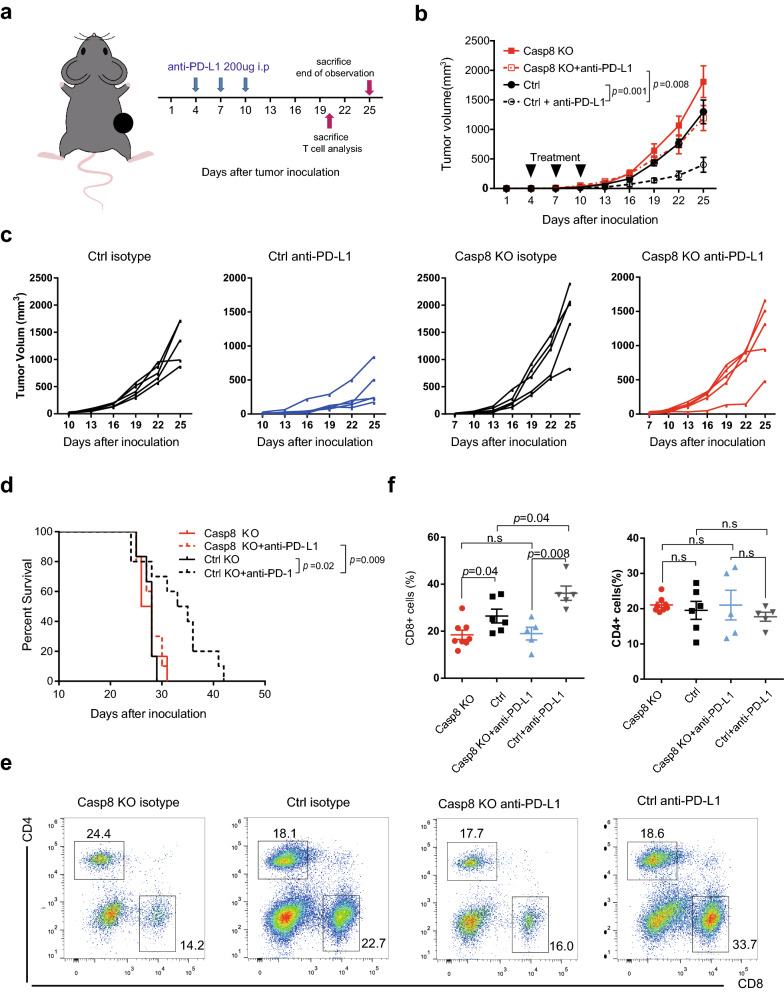
Casp8 knockout induced less CD8^+^ T cell infiltration and poor response to anti-PD-1 treatment in B16F10 mice models. B16-C8KO or control cells (2 × 10^5^) were subcutaneously inoculated into the right flanks of C57BL/6 mice. Mice received 200 µg of intraperitoneal anti-PD-L1 monoclonal antibody or the equivalent isotype control antibody on days 4, 7, and 10. Tumor volume was monitored twice per week. **a** Experimental schematic of the B16F10 mouse model. **b** and **c** Tumor growth curves of the indicated groups (**b**) and each tumor (**c**). **d** The overall survival in the indicated groups. The mice were sacrificed when the tumor volume reached 2000 mm^3^. **e** and **f** CD4^+^ and CD8^+^ tumor-infiltrating cells in subcutaneous xenografts were analyzed by flow cytometry. **e** Representative flow cytometry of tumor-infiltrating T cells pre-gated for viable CD45^+^ cells. **f** Portions of T cells among CD45^+^ leukocytes in tumors

It is well known that the T cell-inflamed gene is a surrogate measure of responsiveness to anti-PD-1/PD-L1 treatment. Since Casp8 knockout significantly reduced CD8^+^ T cell infiltration and the inflammatory response in tumor cells, we speculated that the B16-C8KO clone was resistant to ICB immunotherapy. In the therapeutic mouse model, the anti-PD-L1 antibody was successively injected every three days three times (Fig. 3a). However, this treatment only led to weak (statistically insignificant) tumor control in the B16-C8KO groups compared to their wild-type counterparts (Fig. 3b, c). Accordingly, no survival benefit was observed in B16-C8KO-bearing mice, demonstrating less sensitivity to anti-PD-L1 immunotherapy (Fig. 3d). Furthermore, as observed in the control mice receiving this treatment, anti-PD-L1 administration failed to increase the infiltration of CD8^+^ T cells (Figs. 3e, f and Additional file 1: Fig. S2d).

Taken together, our findings revealed an impaired CD8^+^ T cell response and poor sensitivity to anti-PD-L1 treatment in Casp8 knockout bearing mouse model.

### Responsiveness to ICB treatment in clinical datasets

**Fig. 4:**
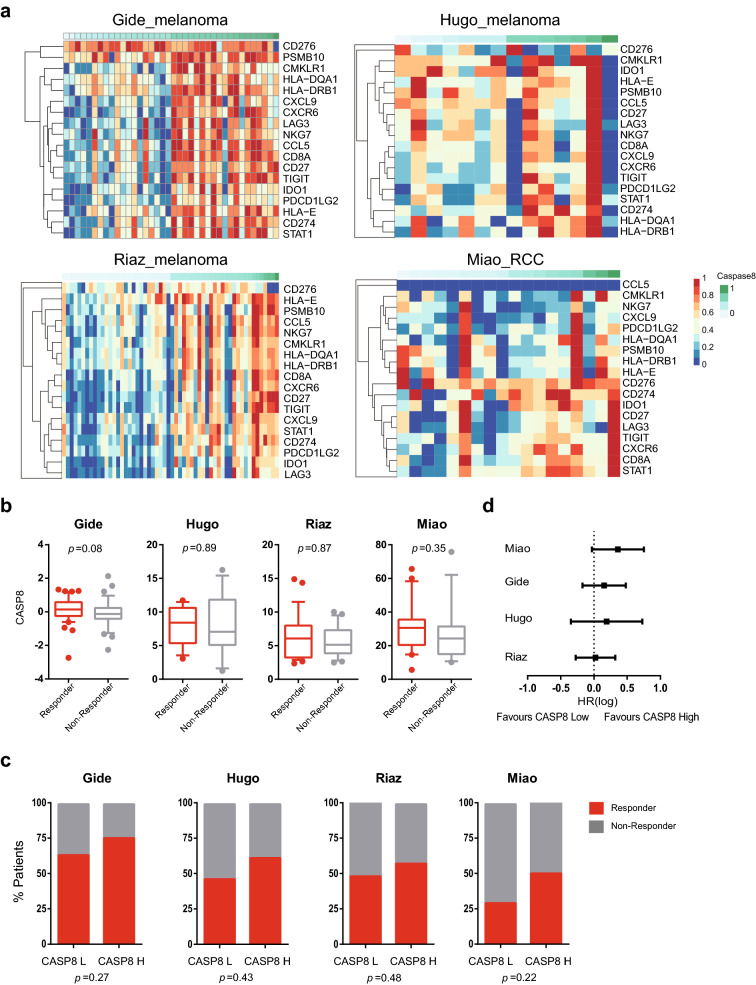
CASP8 expression was related to response to ICB in human cancers. **a** Heatmap of the expression of 18 genes in the T cell-inflamed gene set in four ICB-treated datasets. **b** CASP8 transcription level in responders and non-responders. **c** The response rate in CASP8-high and CASP8-low patients. **d** Forest plot of the hazard ratio for overall survival. The responders included patients with stable disease for more than 6 months, complete response, and partial response according to the Response Evaluation Criteria in Solid Tumors (RECIST 1.1). CASP8 expression lower than the median of each cohort was regarded as CASP8-low; otherwise, expression was regarded as CASP8-high

Subsequently, we aimed to test the clinical relevance of caspase-8 in ICB-treated clinical datasets. To this end, four datasets with available outcome follow-up and baseline RNA sequencing data from tumor biopsies were introduced. Eighteen genes in the T cell-inflamed gene set were used to characterize the immune microenvironment [21–24]. The heatmap displayed enriched expression of signature genes in patients with high levels of CASP8, supporting an immuno-hot microenvironment in these patients (Fig. 4a). Furthermore, to monitor the response to ICB administration, we observed a correlation between CASP8 expression and the responder/non-responder categories in the four datasets (Fig. 4b, c). For the survival outcome, a more favorable overall survival of ICB-treated patients was found in CASP8-high patients than in CASP8-low patients, suggesting a higher sensitivity to ICB treatment in CASP8-high patients (Fig. 4d).

### Impaired antigen presentation and ecto-calreticulin translocation by Casp8 knockout

**Fig. 5:**
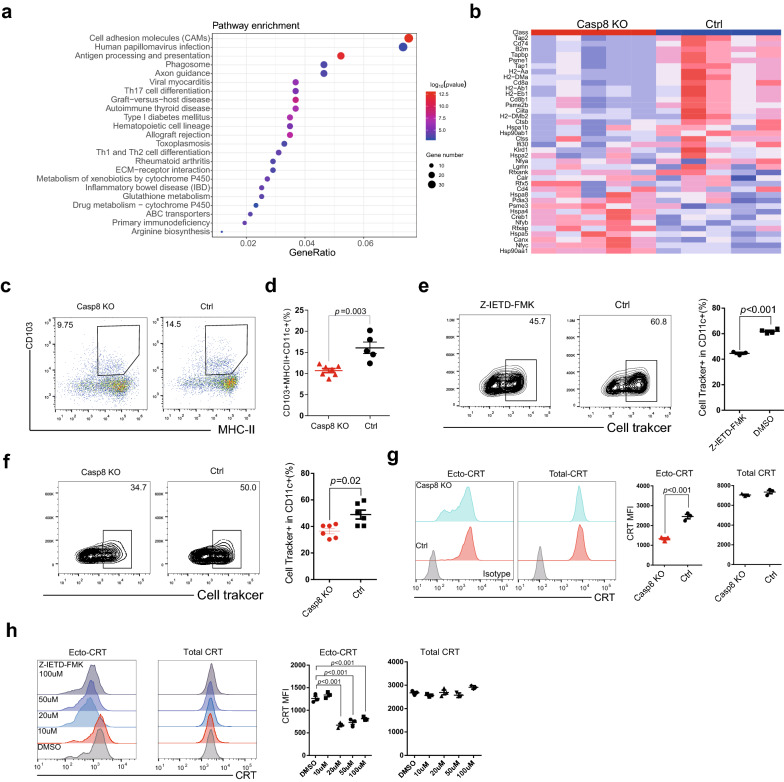
Casp-8 malfunction hampered ecto-CRT transition and induced antigen presentation in B16F10-bearing mice. **a** Kyoto Encyclopedia of Genes and Genomes (KEGG) pathways enriched in genes between Casp8 knockout and control B16F10 cells. Adjusted log10 p-values for the enrichment of KEGG gene sets are presented in color. **b** Heatmap of enriched genes in the lists of Antigen Processing and Presentation genes. **c** CD103^+^MHC-II^+^ dendritic cells in CD11c^+^ cells from drained lymph nodes in the subcutaneous mouse tumor model. **d** Statistical results of CD103^+^MHC-II^+^ cells dendritic cells. **e** and **f** B16F10 cells pre-labeled with the Deep Red dye were treated with Z-IETD-FMK and doxorubicin for 24 h. Labeled tumor cells were injected into the spleens of naive mice. Spleens were collected 2 h after mice received an intrasplenic injection. Splenocytes were subjected to immunostaining with a CD11c antibody to assess phagocytosis. Representative dot plots of flow cytometric analyses depicting the effect of the Casp8 inhibitor Z-IETD-FMK (**e**) and Casp8 knockout (**f**) are shown. Cell surface calreticulin expression (ecto-calreticulin) and total calreticulin in Z-IETD-FMK-treated (**g**) and Casp8 knockout (**h**) B16F10 cells

To explore the underlying mechanism of the Casp8-mediated antitumor immune response, we compared RNA sequencing data to identify the differentially regulated signaling pathways between Casp8-8 knockout and control tumors in a B16F10 tumor-bearing mouse model. Gene Ontology analysis showed that the antigen processing and presentation pathway ranked among the top enriched signaling pathways in tumors with wild-type caspase-8 expression (Figs. 5a and b), leading us to suspect a phenotypic change in dendritic cells in the Casp8 knockout group. Flow cytometry identified significantly fewer antigen-presenting (CD103^+^) dendritic cells from the drained lymph nodes in the Casp8 knockout group than in the control group (Figs. 5c, d and Additional file 1: Fig. S2c). In vivo phagocytosis assays showed that caspase-8 inhibitor treatment (Fig. 5e) or knockout (Fig. 5f) downregulated phagocytosis. Surface-bound calreticulin (ecto-CRT) acts as a bridge for saturable binding sites on phagocytes and favors phagocytosis. In vitro, Casp8 knockout (Fig. 5g) and pharmacological inhibition (Fig. 5h) blocked CRT translocation from the endoplasmic reticulum to the cell surface without altering the total expression of CRT (Figs. 5g, h and Additional file 1: Fig. S4). In addition, Casp8 knockout did not affect other well-known danger-associated molecular patterns, such as HSP70 and HMGB1 (Additional file 1: Fig. S4). In spleen cells with the same pretreatment before phagocytosis, Casp8 malfunction had a limited effect on the ratio of cell death (Additional file 1: Fig. S5). Taken together, our findings suggest a CRT-mediated downregulation of antigen presentation in the Casp8-associated immuno-hot microenvironment.

### Irradiation rescues ecto-CRT and sensitizes Casp8 knockout tumors to ICB treatment

**Fig. 6:**
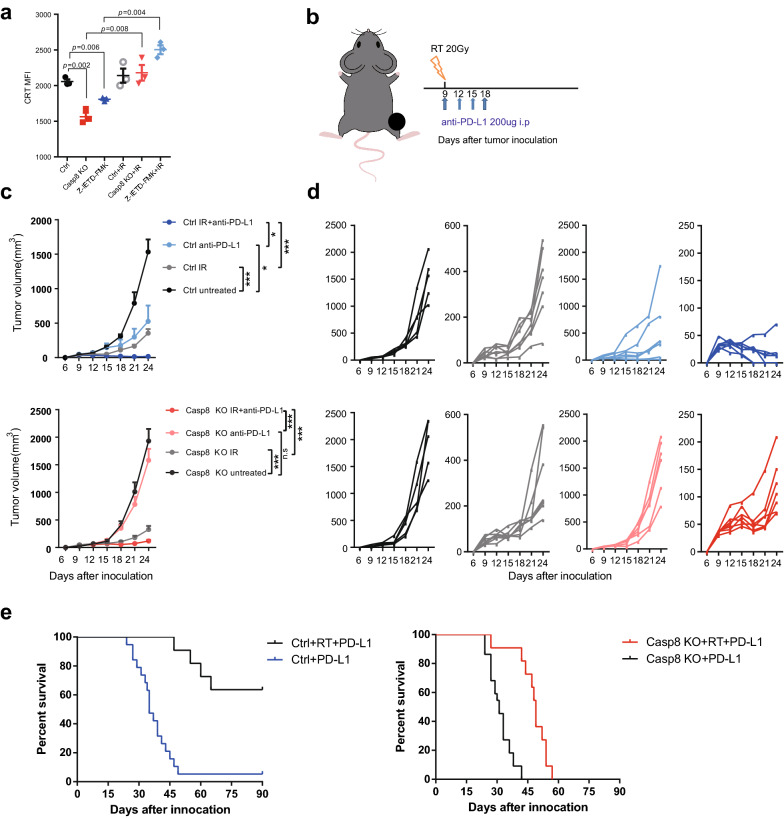
Irradiation rescues ecto-CRT and sensitizes B16F10 tumor cells to ICB in vivo. **a** A single dose of 20 Gy irradiation treatment affected cell surface CRT expression in pre-treated B16F10 cells. **b–e** B16-C8KO or control cells (2 × 10^5^) were subcutaneously inoculated into the right legs of C57BL/6 mice. When the tumors reached approximately 50 mm^3^, the mice were locally irradiated with a single dose of 20 Gy. On the same day, 200 µg of anti-PD-1 monoclonal antibody or the equivalent isotype control antibody was injected intraperitoneally every three days three times. **b** Scheme of irradiation combined with anti-PD-L1 treatment for the B16F10 xenograft mouse model, and tumor growth curves of the indicated groups (**c**) and each tumor (**d**). **e** Overall survival in the indicated groups. When tumor volumes reached 2000 mm^3^, the mice were sacrificed

Radiation can also lead to endoplasmic reticulum stress. We found that ecto-CRT in Casp8 knockout and Casp8 inhibitor-pre-treated B16F10 cells was significantly elevated relative to the control group when a single dose of 20 Gy irradiation was delivered (Fig. 6a). To determine whether radiotherapy could rescue the sensitivity to anti-PD-L1 treatment for Casp8 knockout tumors in vivo, an additional single dose of irradiation was administered on the same day of ICB initiation (Fig. 6b). Consistently, irradiation attenuated poor growth control in the Casp8 knockout group compared to the control group treated with ICB (Fig. 6c, d). In addition, irradiation combined with anti-PD-1 prolonged survival in Casp8 knockout mice compared to those receiving ICB treatment alone, demonstrating the feasibility of a radio-immunotherapy combinational regimen in treating Casp8-deficient patients (Fig. 6e).

## Discussion

ICD can be induced by different stressors such as chemotherapy, irradiation, and targeted anticancer agents. Anthracycline chemotherapy drugs, such as doxorubicin, induce caspase-dependent ICD by emitting damage-associated molecular patterns [27]. Many chemotherapeutic drugs, such as cisplatin, can induce casp8 expression and lead to apoptosis, but these drugs are non-specific inducers [28]. Casp8 is a key regulator of cell death [15]. As both necrosis and pyroptosis are immunogenic, we could infer that a loss of Casp8 function leads to ICD, which triggers a stronger antitumor immune response and benefits ICB therapy. However, our findings suggest that the role of Casp8 is more complex. The clinical data from TCGA and ICB-treated datasets revealed that in certain cancer types, especially melanoma, Casp8 plays a pro-inflammatory role.

Previous studies have revealed that Casp8 may be related to ICB responsiveness, and Casp8 mutant cells accumulated in tumors with a highly cytotoxic TME [29]. One explanation is that a loss of Casp8 function leads to resistance to immune cell death. To test this theory, tumor cells were treated with CRISPR and co-cultured with natural killer cells and T cells [30, 31]. However, caspase-8 knockout was enriched in MC38/MC38-OVA tumors, but not in B16F10 cells, implying that the role of Casp8 may vary among cancer types. In contrast, in MC38-OVA cells co-cultured with OT-I T cells in vitro, treatment with anti-PD-1 failed to enhance tumor eradication [31].

These results imply that the loss of Casp8 function may not be immunogenic, as expected. In clinical samples, high Casp8 expression was related to better overall survival and cytotoxicity of T cells in cancer patients [32]. Furthermore, based on the function of this protein, it is reasonable to infer that this phenomenon occurs because of resistance to inducers of cell death in tumor cells. However, in another study, when the CRISPR-treated tumor cells were treated with cytotoxic T cells, Casp8 knockout tumors were not identified. This indicates that resistance might occur ahead of T cell death [15].

In addition to its role in cell death, our findings showed that Casp8 plays a crucial role in antigen presentation. CRT is a fundamental molecule involved in ICD. When CRT is blocked or knocked down, the immune response is impaired. More precisely, cell-surface-bonded CRT is predominant in ICD. We found that Casp8 knockout downregulated ecto-CRT in our B16F10 model, which is consistent with previous studies [33]; Casp8 knockout mice also showed a weak antitumor response in the CT26 mouse model. These reports support our finding that Casp8 is a key regulator of ecto-CRT, which affects the response to ICB. However, in the endoplasmic reticulum stress model, ecto-CRT was not Casp8-dependent, and a similar finding was reported in the photodynamic therapy model [34]. In clinical practice, the detection of Casp8 mutations is practical, implying that such mutations might be independent predictive markers of the response to ICB.

Furthermore, we tried to rescue the resistance to ICB caused by the Casp8 mutation. Irradiation is an important therapeutic method used to overcome low responsiveness to ICB in clinical practice. As far as immunity was concerned, irradiation was thought to have a dual effect, both inhibiting and promoting immunity [35, 36]. Lamerton verified that if the whole body of the animals was exposed to radiation of 1.76 Gy/day or 0.84 Gy/day, their immune system first responded positively and peripheral blood count increased; however, within 20 days, their bone marrow failed to produce platelets and leukocytes, and their immune system was destroyed [37], ultimately resulting in death. However, an increasing number of studies have shown that local irradiation might enhance antitumor immunity; for example, 8.5 Gy × 5 irradiation of tumors was reported to enhance MHC class I expression and dendritic cell function and improve the efficacy of tumor immunotherapy [38, 39]. High LET/RBE irradiation, such as particle and heavy-ion radiation, could induce single- and double-strand DNA breaks [40]. Meanwhile, in living tissues generating ROS/RNS and H_2_O_2_, irradiation damages DNA, proteins, and membranes, resulting in new antigen production and strengthening of the immune response. A previous study by our group confirmed that a single local irradiation dose of 20 Gy for tumors could enhance the number of tumor-infiltrating CD8^+^ CTLs in the TME of B16F10 tumors, and the depletion of CD8^+^ T cells significantly weakened the therapeutic effect of irradiation [41]. In this study, irradiation combined with ICB improved the ORR and prolonged progression-free and overall survival. In our model, we found that in Casp8-deficient patients, radiation might be an effective approach for overcoming ICB resistance. We explained a new mechanism where stereotactic body radiation therapy enhances ICB therapy by inducing calreticulin expression through caspase-8 inhibition, thus enriching the immunological theory of stereotactic body radiation therapy-enhanced immunotherapy.

## Conclusions

Casp8 deficiency, knockout, and inhibitor treatment led to an impaired ecto-calreticulin transition, which in turn resulted in hampered antigen presentation and a cold TME, which are traits associated with ICB resistance. Irradiation could rescue the ecto-calreticulin expression of tumor cells and improve phagocytosis to overcome ICB resistance. Consistent with TCGA and ICB-treated cohorts, Casp8 expression was correlated with an inflamed TME and a better response to ICB. These results imply that patients with Casp8 loss-of-function mutations may not benefit from ICB alone; however, a radiation combination strategy might sensitize non-responders and improve clinical outcomes.

## Supplementary Information

**Additional file 1:** **Figure S1.** Validation the construction of casp8-KO cell line. (**a**) sequencing show the nucleotide sequence of casp8-KO B16F10 cell line. (**b**) Western blotting identified the efficacy of casp8 knockout at protein level. (**c, d**) TUNEL assay and statistical summary. **Figure S2.** The gating strategy for the FACS analyses. (**a** , **b** and **c**) B16-C8KO or control cells were subcutaneously inoculated into the right flanks of C57BL/6 mice, tumor microenvironment of subcutaneous xenografts was analyzed. The gating strategy for flow cytometry of CD4^+^ or CD8^+^ tumor-infiltrating cells (**a**), GZMB and INF-γ produced by CD8^+^ T cells (**b**) and the CD103^+^MHC-II^+^ dendritic cells in CD11c^+^ cells from tumor draining lymph nodes (**c**). 2 × 10^5^ B16-C8KO or control cells were subcutaneously inoculated into the right flanks of C57BL/6 mice. Mice received 200 µg intraperitoneal anti-PD-L1 monoclonal antibody or the equivalent isotype control antibody on days 4, 7, and 10. The gating strategy for flow cytometry of CD4^+^ or CD8^+^ tumor-infiltrating cells in subcutaneous xenografts (**d**). **Figure S3.** Tumor-associated macrophages and myeloid-derived suppressor cells in tumor microenvironment. (**a** and **b**) Representative flow cytometry of F4/80^+^CD11b^+^(**a**) and Gr-1^+^ CD11b^+^ (**b**) tumor-infiltrating cells (gate in CD45^+^ cells). (**c**) Fractions of F4/80^+^CD11b^+^ and Gr-1^+^ CD11b^+^ cells in CD45^+^ leukocytes in tumors. **Figure S4.** Protein level of DAMPs. (**a**) ELISA assesses the concentration of HMGB1 in condition medium from control and casp8-KO cell lines. (**b**) Western blotting showed the protein level of DAMPs. **Figure S5.** Doxorubicin induced B16F10 cell death. (**a**) The indicated B16-C8KO and control cells were treated with 25 µM doxorubicin for 24 h. Then cells were harvest and stained with APC Annexin V Apoptosis Detection Kit with PI (640932, biolegend) following the manufacturer’s instructions, then cell viability was detected by flow cytometry.

## Data Availability

The datasets supporting the conclusions in the cohort analysis of this article are available from TCGA datasets and published articles.

## Abbreviations

ANOVA: Analysis of variance
ICB: Immune checkpoint blockade
ICD: Immunogenic cell death
GSEA: Gene Set Enrichment Analysis
KEGG: Kyoto Encyclopedia of Genes and Genomes
PD-1: Programmed death-1 receptor
PD-L1: Programmed death ligand 1
TME: Tumor microenvironment
TCGA: The Cancer Genome Atlas
CRT: Calreticulin
ecto-CRT: Surface-bound calreticulin
