# Supplementary material for: Caspase-8 contributes to an immuno-hot microenvironment by promoting phagocytosis via an ecto-calreticulin-dependent mechanism
Source: Exp Hematol Oncol. 2023 Jan 12;12:7. doi: 10.1186/s40164-022-00371-1 (PMC9835222; doi:10.1186/s40164-022-00371-1)
Supplement: Supplementary file 1 — Additional file 1: Figure S1. Validation the construction of casp8-KO cell line. (a) sequencing show the nucleotide sequence of casp8-KO B16F10 cell line. (b) Western blotting identified the efficacy of casp8 knockout at protein level. (c, d) TUNEL assay and statistical summary. Figure S2. The gating strategy for the FACS analyses. (a , b and c) B16-C8KO or control cells were subcutaneously inoculated into the right flanks of C57BL/6 mice, tumor microenvironment of subcutaneous xenografts was analyzed. The gating strategy for flow cytometry of CD4+ or CD8+ tumor-infiltrating cells (a), GZMB and INF-γ produced by CD8+ T cells (b) and the CD103+MHC-II+ dendritic cells in CD11c+ cells from tumor draining lymph nodes (c). 2 × 10^5^ B16-C8KO or control cells were subcutaneously inoculated into the right flanks of C57BL/6 mice. Mice received 200 µg intraperitoneal anti-PD-L1 monoclonal antibody or the equivalent isotype control antibody on days 4, 7, and 10. The gating strategy for flow cytometry of CD4+ or CD8+ tumor-infiltrating cells in subcutaneous xenografts (d). Figure S3. Tumor-associated macrophages and myeloid-derived suppressor cells in tumor microenvironment. (a and b) Representative flow cytometry of F4/80+CD11b+(a) and Gr-1+ CD11b+ (b) tumor-infiltrating cells (gate in CD45+ cells). (c) Fractions of F4/80+CD11b+ and Gr-1+ CD11b+ cells in CD45+ leukocytes in tumors. Figure S4. Protein level of DAMPs. (a) ELISA assesses the concentration of HMGB1 in condition medium from control and casp8-KO cell lines. (b) Western blotting showed the protein level of DAMPs. Figure S5. Doxorubicin induced B16F10 cell death. (a) The indicated B16-C8KO and control cells were treated with 25 µM doxorubicin for 24 h. Then cells were harvest and stained with APC Annexin V Apoptosis Detection Kit with PI (640932, biolegend) following the manufacturer’s instructions, then cell viability was detected by flow cytometry. [file 40164_2022_371_MOESM1_ESM.docx]

**Additional file 1**

**Figure S1. Validation the construction of casp8-KO cell line.**


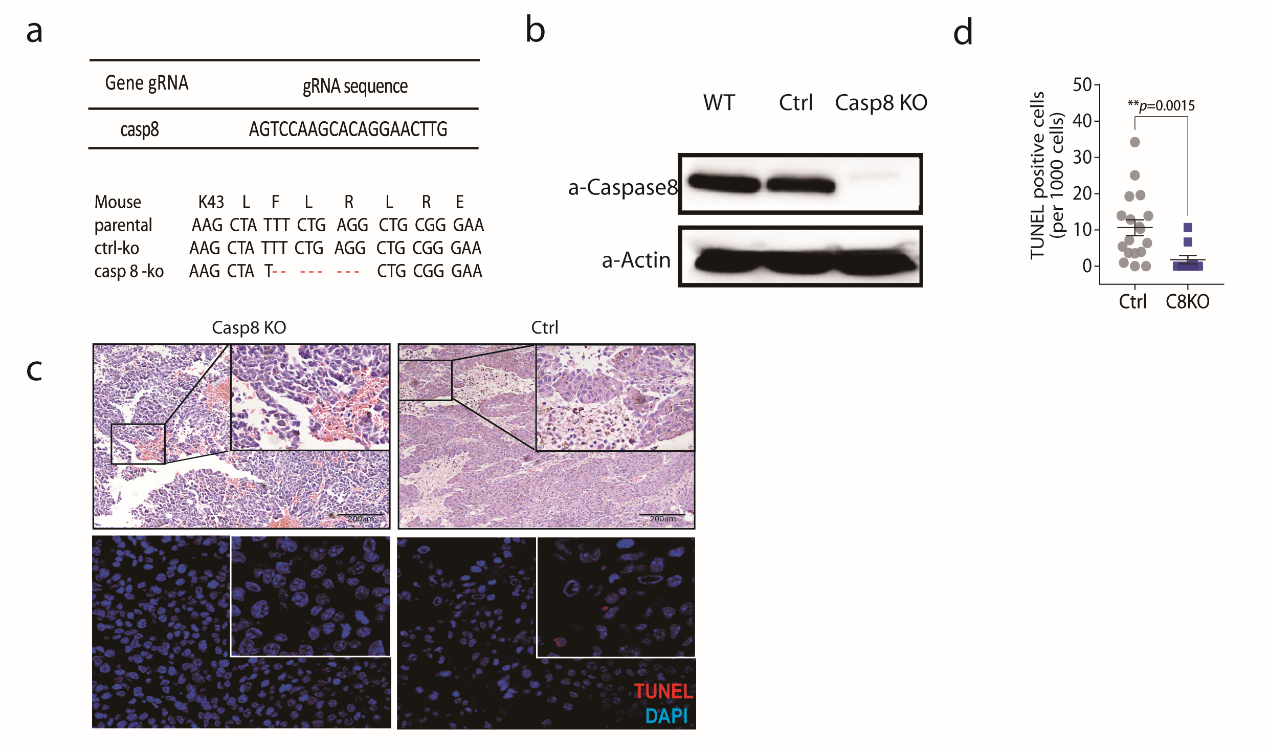


1. sequencing show the nucleotide sequence of casp8-ko B16F10 cell line. **(b)** Western blot identify the efficacy of casp8 knock out at protein level. **(c, d)** TUNEL assay and statistical summary.

**Figure S2. The gating strategy for the FACS analyses.**


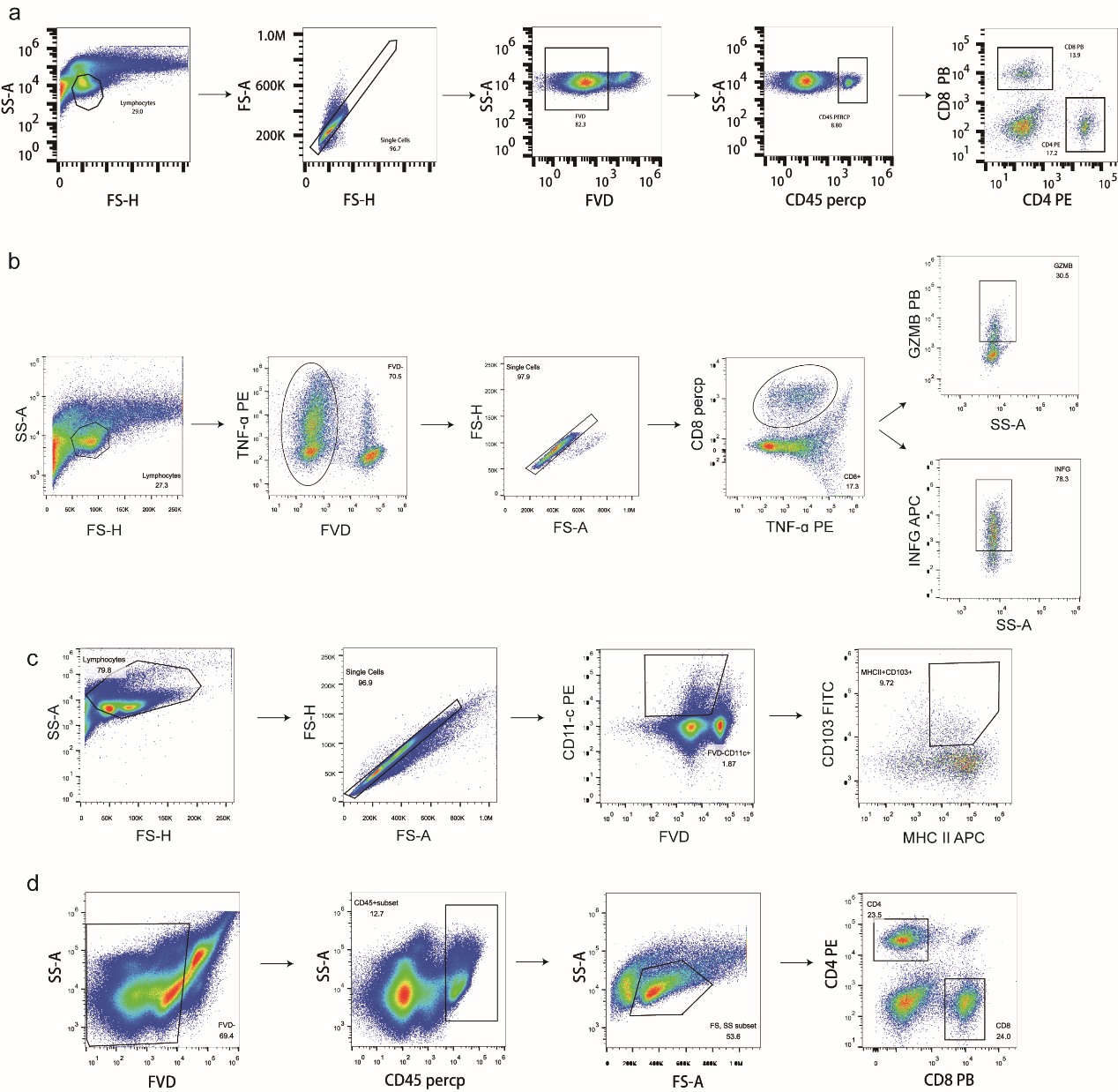


**(a**, **b and c)** B16-C8KO or control cells were subcutaneously inoculated into the right flanks of C57BL/6 mice, tumor microenvironment of subcutaneous xenografts was analyzed. The gating strategy for flow cytometry of CD4+ or CD8+ tumor-infiltrating cells (**a**), GZMB and INF-γ produced by CD8+ T cells (**b**) and the CD103^+^MHC-II^+^ dendritic cells in CD11c^+^ cells from tumor draining lymph nodes (**c**). 2 × 10^5^ B16-C8KO or control cells were subcutaneously inoculated into the right flanks of C57BL/6 mice. Mice received 200 µg intraperitoneal anti-PD-L1 monoclonal antibody or the equivalent isotype control antibody on days 4, 7, and 10. The gating strategy for flow cytometry of CD4+ or CD8+ tumor-infiltrating cells in subcutaneous xenografts (**d**).

**Figure S3. Tumor-associated macrophages and myeloid-derived suppressor cells in tumor microenvironment.**


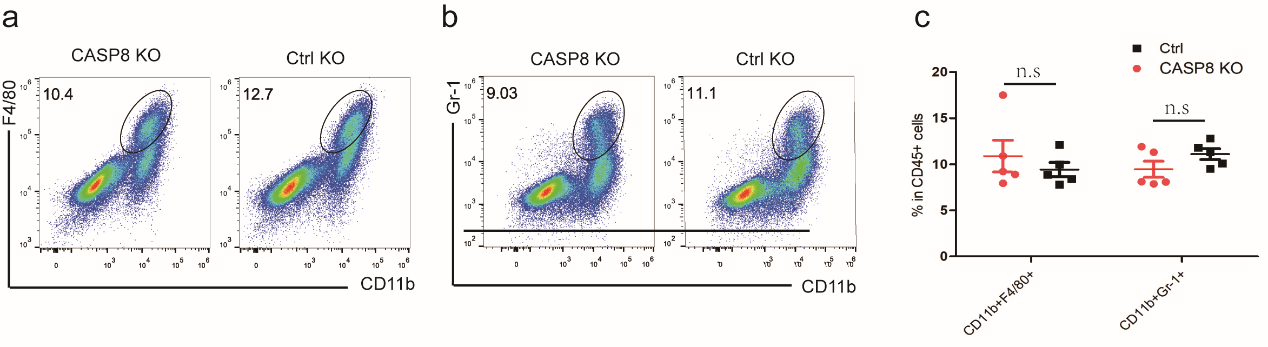


**(a** and **b)** Representative flow cytometry of F4/80+CD11b+(A) and Gr-1+ CD11b+ (B) tumor-infiltrating cells (gate in CD45+ cells). **(c)** Fractions of F4/80+CD11b+(A) and Gr-1+ CD11b+ cells in CD45^+^ leukocytes in tumors

**Figure S4. Protein level of DAMPs**


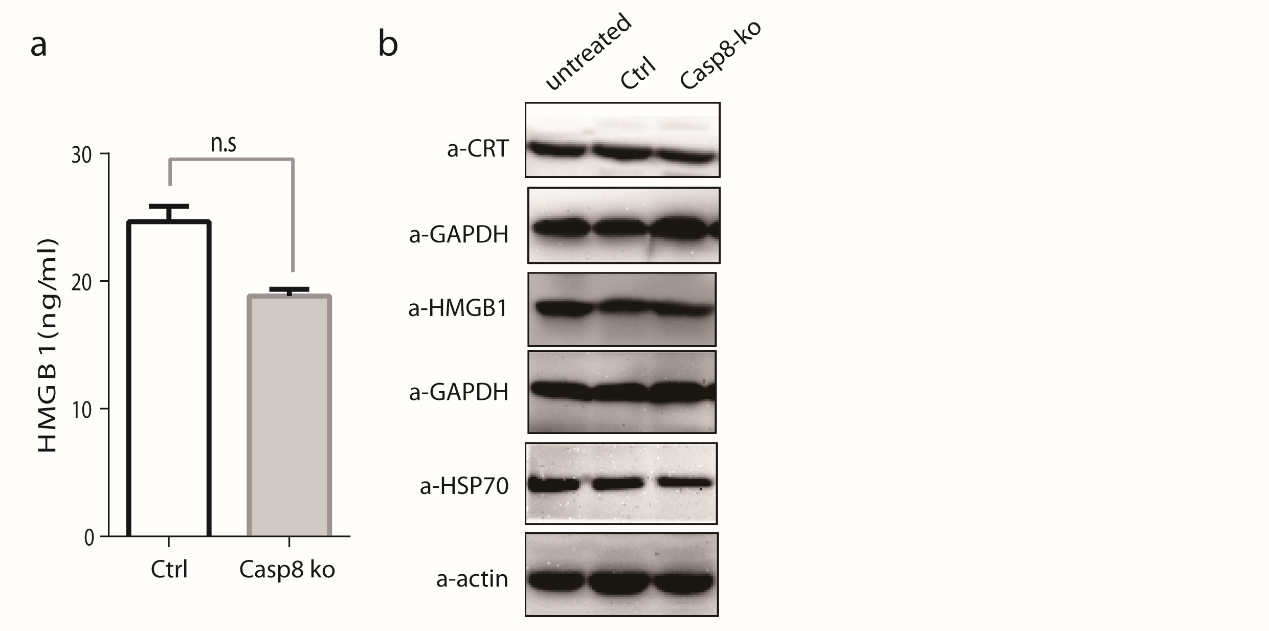


1. ELISA assesses the concentration of HMGB1 in condition medium from control and casp8-KO cell lines. **(b)** Western blot shows the protein level of DAMPs.

**Figure S5. Doxorubicin induced B16F10 cell death**


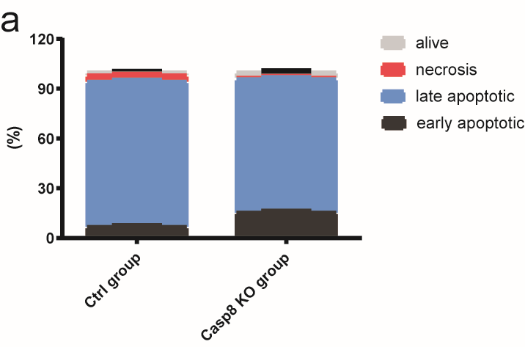


1. The indicated B16-C8KO and control cells were treated with 25 µM doxorubicin for 24 h. Then cells were harvest and stained with APC Annexin V Apoptosis Detection Kit with PI (640932, biolegend) following the manufacturer’s instructions, then cell viability was detected by flow cytometry.
